# *Synechococcus elongatus* as a model of photosynthetic bioreactor for expression of recombinant β-glucosidases

**DOI:** 10.1186/s13068-019-1505-9

**Published:** 2019-07-03

**Authors:** Raíza Azevedo, Jéssika Lawall Lopes, Manuel Macedo de Souza, Betania Ferraz Quirino, Letícia Jungmann Cançado, Luis Fernando Marins

**Affiliations:** 1 Laboraty of Molecular Biology, Institute of Biological Sciences (ICB), Federal University of Rio Grande (FURG), Av. Itália, Km 8, Rio Grande, RS 96203-900 Brazil; 2 Institute of Oceanography (IO), Federal University of Rio Grande (FURG), Av. Itália, Km 8, Rio Grande, RS 96203-900 Brazil; 3 Embrapa-Agroenergy, Parque Estação Biológica, s/n, Brasília, DF 70770-901 Brazil

**Keywords:** Cellulases, Cyanobacteria, pET system, Genetic engineering, Heterologous expression

## Abstract

**Background:**

The production of glucose from cellulose requires cellulases, which are obtained from decomposing microorganisms such as fungi and bacteria. Among the cellulases, β-glucosidases convert cellobiose to glucose and have low concentration in commercial cocktails used for the production of second-generation (2G) ethanol. Genetic engineering can be used to produce recombinant β-glucosidases, and cyanobacteria may be interesting bioreactors. These photosynthetic microorganisms can be cultured using CO_2_ emitted from the first-generation ethanol (1G) industry as a carbon source. In addition, vinasse, an effluent of 1G ethanol production, can be used as a source of nitrogen for cyanobacteria growth. Thus, photosynthetic bioreactors cannot only produce cellulases at a lower cost, but also reduce the environmental impact caused by residues of 1G ethanol production.

**Results:**

In the present work, we produced a strain of *Synechococcus elongatus* capable of expressing high levels of a heterologous β-glucosidase from a microorganism from the Amazonian soil. For this, the pET system was cloned into cyanobacteria genome. This system uses a dedicated T7 RNA polymerase for the expression of the gene of interest under the control of a nickel-inducible promoter. The results showed that the pET system functions efficiently in *S. elongatus*, once nickel induced T7 RNA polymerase expression which, in turn, induced expression of the gene of the microbial β-glucosidase at high levels when compared with non-induced double transgenic strain. β-glucosidase activity was more than sevenfold higher in the transformed cyanobacteria than in the wild-type strain.

**Conclusions:**

The T7 system promotes high expression levels of the cloned gene in *S. elongatus*, demonstrating that the arrangement in which an exclusive RNA polymerase is used for transcription of heterologous genes may contribute to high-level gene expression in cyanobacteria. This work was the first to demonstrate the use of cyanobacteria for the production of recombinant β-glucosidases. This strategy could be an alternative to reduce the release of 1G ethanol by-products such as CO_2_ and vinasse, not only contributing to decrease the cost of β-glucosidase production, but also mitigating the environmental impacts of ethanol industrial plants.

## Background

Concerns about world energy consumption and the need to reduce the emission of greenhouse gases have accelerated scientific research on new energy sources and/or more efficient use of available feedstocks. Agroindustrial by-products of vegetal origin have great potential for renewable and sustainable energy generation due to their abundance, biodegradability, hydrocarbon content and low-cost/benefit ratio [1]. One such example is sugarcane bagasse, a by-product of sugarcane-based bioethanol industry, with an annual estimated production of 540 Mt [2]. Its approximate composition is cellulose (50%), hemicellulose (25%) and lignin (25%) [3]. Although much of the bagasse produced is burned in the boilers for bioelectricity cogeneration for the industrial facility itself, studies show that the use of lignocellulosic biomass for the production of second-generation (2G) ethanol may present a lower risk and greater profitability for the investors [4]. Integration of 2G ethanol production into already existing 1G ethanol biorefineries may be an attractive business [5] because of the possibility of (1) increasing ethanol production by 30%, without the need to expand the sugarcane-planted area; (2) reducing CO_2_ emissions during the production process when compared to 1G ethanol, (3) using the existing and consolidated logistics infrastructure for distribution and utilization (the same used for 1G ethanol production); (4) using by-products and wastewater from the production of 1G ethanol; and (5) production during the off-season of sugarcane [6, 7].

Second-generation (2G) ethanol is produced in four stages: pre-treatment, hydrolysis, fermentation and distillation. In general, the process consists in the removal of lignin and reduction in the degree of cellulose crystallization (pre-treatment) to facilitate the enzymatic hydrolysis by cellulases and β-glucosidases, which act to convert cellulose into glucose. Subsequently, the glucose generated is fermented into ethanol by yeast [1]. Although 2G ethanol has a better energy balance than 1G ethanol and it is environmentally “friendlier”, its production is still not economically viable due to the high cost of the pre-treatment and enzymatic hydrolysis steps [8, 9].

Cellulose saccharification (hydrolysis) is catalyzed by three types of enzymes: endoglucanases (EC 3.2.1.4), exoglucanases (EC 3.2.1.91) and β-glucosidases (EC 3.2.1.21). Endoglucanases break the inner bonds of the cellulose, reducing its degree of polymerization and exposing the microfibrils to the action of the exoglucanases at the ends of the chains, thus releasing glucose and cellobiose. The produced cellobiose acts as inhibitor of the endo- and exoglucanases, negatively affecting enzymatic hydrolysis. Therefore, β-glucosidases have a crucial role in the process. As they convert cellobiose to glucose, they not only make glucose available for yeast fermentation, but also they prevent cellulase inhibition by cellobiose. Although the action of the β-glucosidases represents the critical point of the enzymatic hydrolysis of the cellulose, its concentration in the currently commercialized enzymatic cocktails is low, increasing the cost of 2G ethanol production [10]. Finding organisms able to produce large amounts of cellulose deconstruction enzymes has been an obstacle to the generation of 2G ethanol [11]. Through genetic engineering, it is possible to develop biofactories for the production of recombinant proteins with desirable characteristics. Some genetically modified organisms have been shown to be more efficient in the production of β-glucosidases than non-transgenic organisms traditionally used in the production of enzymes [10]. For example, a *Pichia pastoris* strain was genetically engineered for the expression of a novel β-glucosidase (NpaBGS) isolated from the fungus *Neocallimastix patriciarum*, which showed better performance than commercial Novozym − 188 β-glucosidase (Novozymes) [11].

Cyanobacteria are considered attractive hosts for the production of recombinant proteins due to their photosynthetic efficiency (superior to terrestrial plants) [12], cosmopolitan distribution, minimum growth requirements (basically solar energy, water and CO_2_), short life cycle, easy genetic manipulation and high biomass productivity compared to agricultural crops [13, 14]. Genetic engineering has been widely employed in cyanobacteria for the development of biofactories to produce molecules of commercial interest at a reduced cost. Functional enzymes such as ketoacid decarboxylase from *Lactococcus lactis*, used to produce isobutyraldehyde and isobutanol [15], isoprene synthase from *Pueraria montana*, for isoprene generation [16] and larvicidal proteins from *Bacillus thuringiensis* against the mosquito *Aedes aegypti* [17] are some examples of such enzymes successfully produced in cyanobacteria.

To improve the yield of biomolecules in cyanobacteria, several vectors for chromosome integration, called SyneBricks, have been described for expression of heterologous genes in *Synechococcus elongatus* PCC 7942 [18]. One of the proposed expression systems was based on the pET expression system developed for expression of heterologous genes in *Escherichia coli* [19]. This system consists of an insertion of the bacteriophage T7 RNA polymerase into the cyanobacteria genome under the control of an inducible promoter. T7 RNA polymerase binds with high specificity to the T7 promoter (P_T7_), thus triggering targeted transcription of the heterologous gene cloned downstream of this promoter. With such an expression platform, it is not only possible to increase the production of recombinant proteins, but also to control expression of the gene of interest, yielding the protein only when desired.

It has been estimated that recombinant protein production in cyanobacteria enables conversion of more than 50% of atmospheric CO_2_ into protein biomass [20]. In addition to CO_2_, other residues of the sugarcane-derived 1G ethanol production can be used as a source of nutrients to further contribute to a sustainable and low-cost production of enzymes used in the 2G ethanol industry. Vinasse, a nitrogen-rich effluent derived from alcoholic fermentation, can be used in the cultivation of cyanobacteria for biofuel (e.g., biodiesel) production [21]. In this context, this study aimed to transform the cyanobacteria *Synechococcus elongatus* PCC 7942 into a photosynthetic bioreactor for the production of recombinant β-glucosidases. Due to the fact that CO_2_ and vinasse can be used for *Synechococcus elongatus* biomass production, it is possible to integrate the 1G and 2G ethanol processes as previously suggested by Brasil et al. [5].

## Results and discussion

### Construction of pET expression system and insertion into *S. elongatus* PCC 7942

Studier and Moffatt developed a gene expression system based on the use of an exclusive RNA polymerase for the expression of heterologous genes [19]. Known as pET expression system, this protein expression platform was developed by inserting the T7 bacteriophage RNA polymerase gene into the *Escherichia coli* genome. T7 RNA polymerase is characterized by having highly selective and efficient transcription due to high specific binding to the T7 promoter, which does not occur naturally in the host cell genome. Thus, this platform is widely employed for heterologous protein expression in *E. coli*. In the present work, we used the pET system for expression of a cellulolytic enzyme in *S. elongatus* PCC 7942.

Bergmann et al. [22] constructed a metagenomic library to isolate new β-glucosidases from the microbial community of Amazonian soil. Two prokaryotic genes belonging to the GH3 and GH1 families (AMBGL17 and AMBGL18, respectively) were isolated. Although there is no evidence that AMBGL17 gene codes for a true cellobiase, it has been functionally considered as one because its ORF region codes for a β-glucosidase belonging to GH3 family. As the enzymatic activity of AMBGL17 has already been biochemically characterized and revealed that this enzyme not only possesses a high enzymatic activity and affinity with the substrate (*V*_max_: 16 mM s^−1^, *K*_m_: 0.30 ± 0.017 mM, *K*_cat_: 38.57 s^−1^) but also a higher catalytic efficiency (*K*_cat_/*K*_m_) than many β-glucosidases described in the literature (128.56 s^−1^ mM^−1^), it was chosen for the present work [22].

**Fig. 1:**
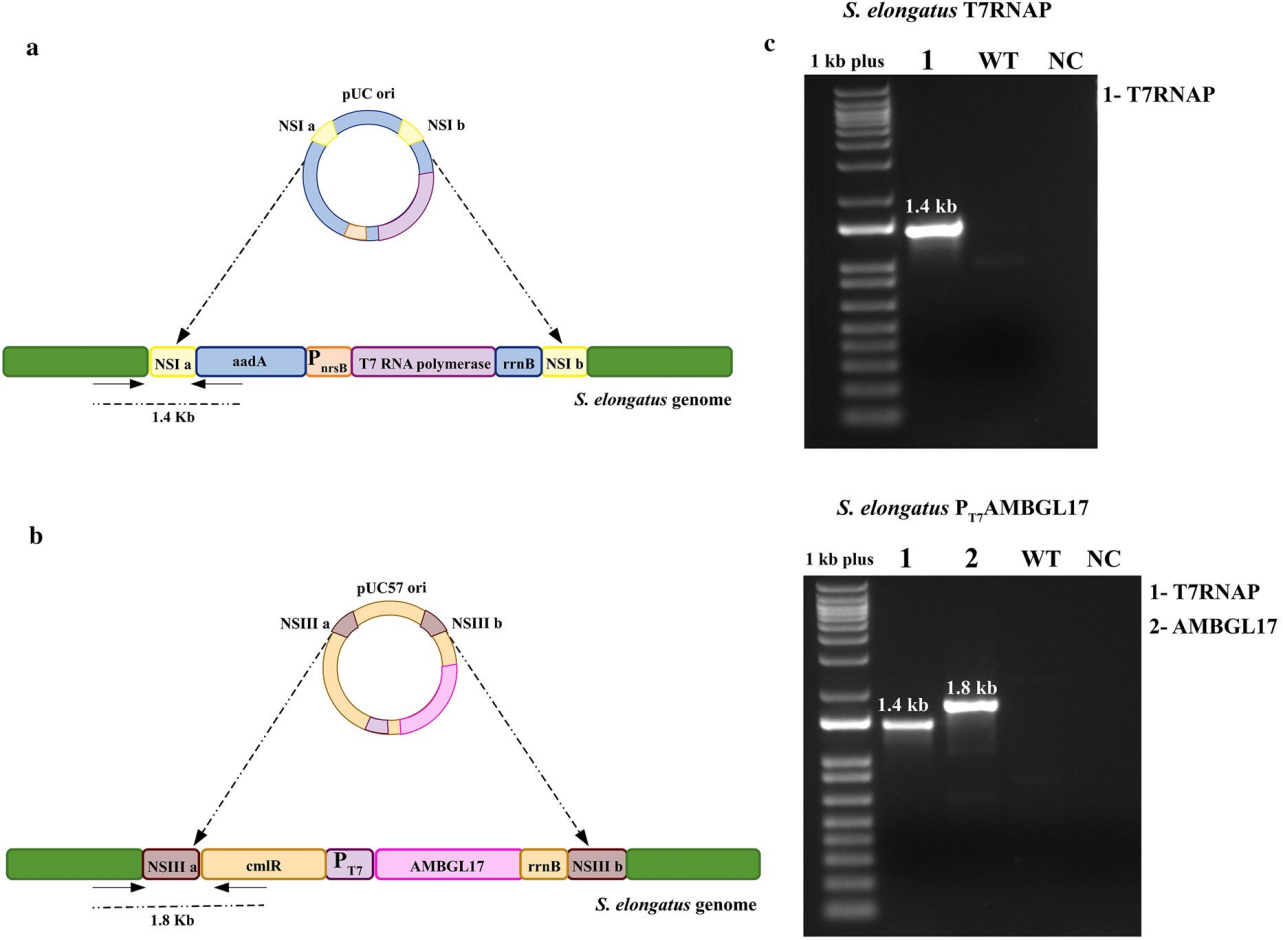
Characteristics of expression cassettes and confirmation of integration of the pSyn_T7RNAP and pHN1_AMBGL17 vectors into the genome of *Synechococcus elongatus* PCC 7942. **a** pSyn_T7RNAP: Homologous recombination site (NSI a and NSI b), spectinomycin resistance gene (aadA), promoter region (P_nrsB_), T7 RNA polymerase, transcription termination region (rrnB). **b** pHN1_AMBGL17: Homologous recombination site (NSIII a and NSIII b), chloramphenicol resistance gene (cmlR), promoter region (P_T7_), β-glucosidase (AMBGL17), transcription termination region (rrnB). Dashed arrows indicate annealing position of the primers with respective sizes. **c** Confirmation of genomic integration by PCR. WT: *S. elongatus* wild type, NC: negative control reaction (without DNA template), 1 kb plus: 1 Kb Plus DNA Ladder

For the construction of the pET expression system, two neutral sites (NSI and NSIII) were used for the insertion of genes into the cyanobacterial genome through homologous recombination [23]. The gene encoding T7 RNA polymerase (T7RNAP) was inserted at neutral site 1 (NSI) under control of the Ni^2+^-inducible P_nrsB_ promoter (Fig. 1a). Insertion of the T7RNAP expression cassette into the cyanobacterial genome was confirmed by PCR with the amplification of a 1.4-kb fragment, which corresponds to the distance between the genomic locus Synpcc7942_2499 (flotillin membrane protein; flanking the NSI site) and the spectinomycin resistance gene (aadA gene: aminoglycoside resistance protein AadA) present in the genetic construct (Fig. 1a, c). After confirmation of T7RNAP gene insertion into the cyanobacterial genome, a second transformation was performed with the pHN1_AMBGL17 plasmid (Fig. 1b) for integration of the β-glucosidase gene (AMBGL17) at neutral site 3 (NSIII). Integration was confirmed by the amplification of a 1.8-kb fragment corresponding to the distance between the genomic locus Synpcc7942_0741 (Phage tail protein I gene) and the chloramphenicol resistance gene (cmlR gene: chloramphenicol resistance protein) present in the second genetic construct (Fig. 1b, c).

The double-transformed colonies called *S. elongatus* P_T7_AMBGL17 were confirmed by the amplification of a 1.8-kb DNA fragment consisting of part of the genomic locus Synpcc7942_0741 (Phage tail protein I) and part of the chloramphenicol resistance gene present in the genetic construct (Fig. 1c). Vector choice (integrative or replicative) for genetic manipulation is essential to ensure successful transformation. In this work, we chose to use integrative vectors to guarantee gene stability through recombination, with long-term maintenance of the transgenic lineage, as suggested by Heidorn et al. [24].

### Effects of transgenesis and nickel addition on cell growth rates

**Fig. 2:**
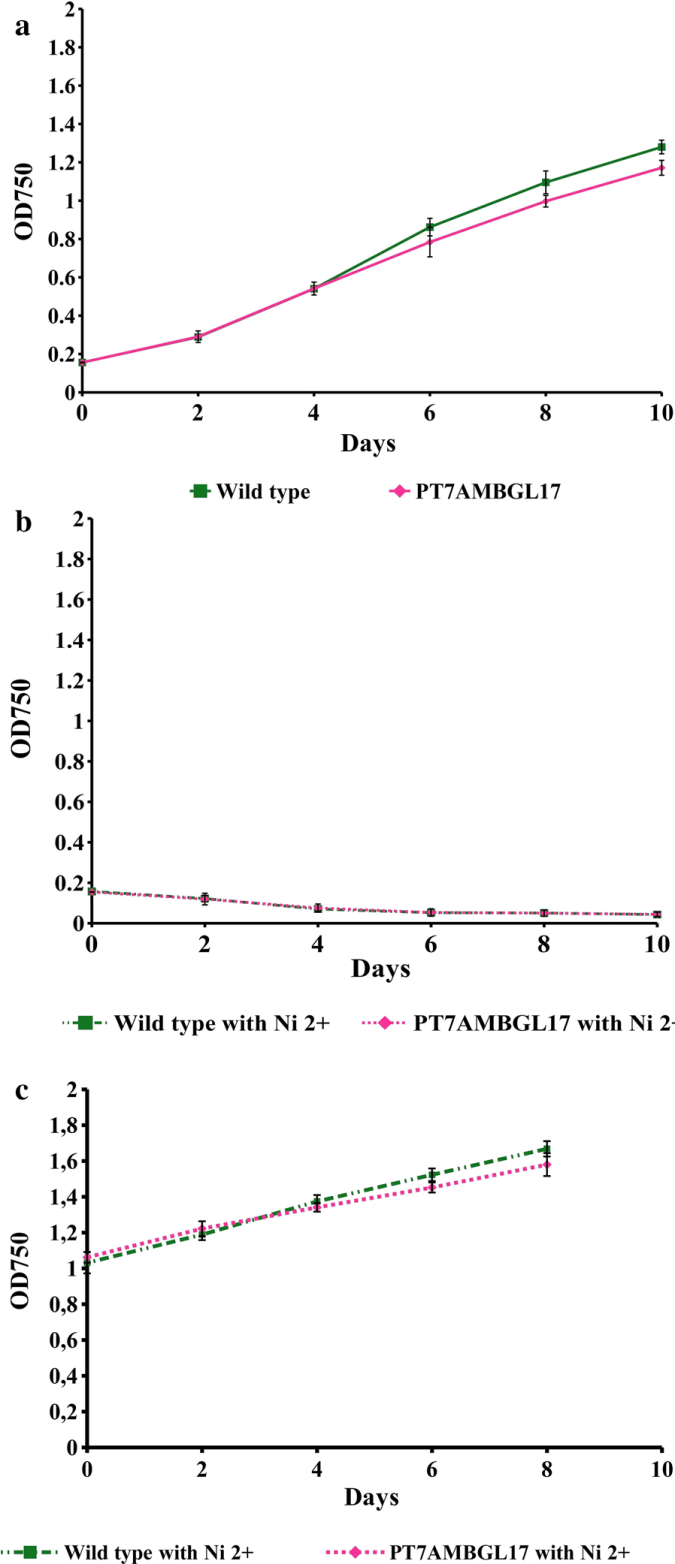
*Synechococcus elongatus* PCC 7942 growth curves. **a** Effects of genome integration of the two expression cassettes over the transgenic *S. elongatus* (P_T7_AMBGL17) growth rate in comparison with wild-type growth rate. **b** Effects of 5 μM nickel (Ni^2+^) addition to *S. elongatus* wild-type and transformed cultures during the Lag phase. **c** Effects of 5 μM nickel (Ni^2+^) addition to *S. elongatus* wild-type and transformed cultures during the stationary phase

As *Synechococcus elongatus* PCC 7942 may harbor from 1 to 10 genome copies, we cannot guarantee that heterologous genes (T7RNAP and AMBGL17) integrated in all genome copies, being virtually impossible to get a homogeneous expression among different strains. Besides that, those genes can be lost during cell division, even in the presence of a selective agent. Due this fact, we performed a qPCR screening 10 strains of double transgenic cyanobacterium (P_T7_AMBGL17) to evaluate the difference of gene expression among them and select the strain that presented the best level of expression to be used in the further experiments (data not shown). No significant statistical differences in growth were observed when wild-type and double transgenic (P_T7_AMBGL17) strains were compared, indicating that foreign DNA integration did not affect cyanobacteria growth (Fig. 2a). However, addition of 5 μM Ni^2+^ in the latency phase (Lag) had a toxic effect to both transformed and wild-type cells, leading to mortality of both strains (Fig. 2a). Lag phase is a period when cyanobacteria adapt to the environmental conditions and is characterized by little or no cell division, but intense metabolic activity [25]. The visible toxic effects that resulted from nickel addition at this stage of cultivation suggest that nickel efflux pumps were not activated, probably causing nickel to accumulate inside the cell, leading to the observed toxic effect. At the stationary phase, when metabolic activity decreases and cell division increases, nickel did not affect cellular viability, demonstrating that the activation of the pET system is not toxic for the wild-type or the double transgenic strains (Fig. 2c). Based on these results, induction at the stationary phase was used in all subsequent experiments.

### Determination of expression levels from T7 system

**Fig. 3:**
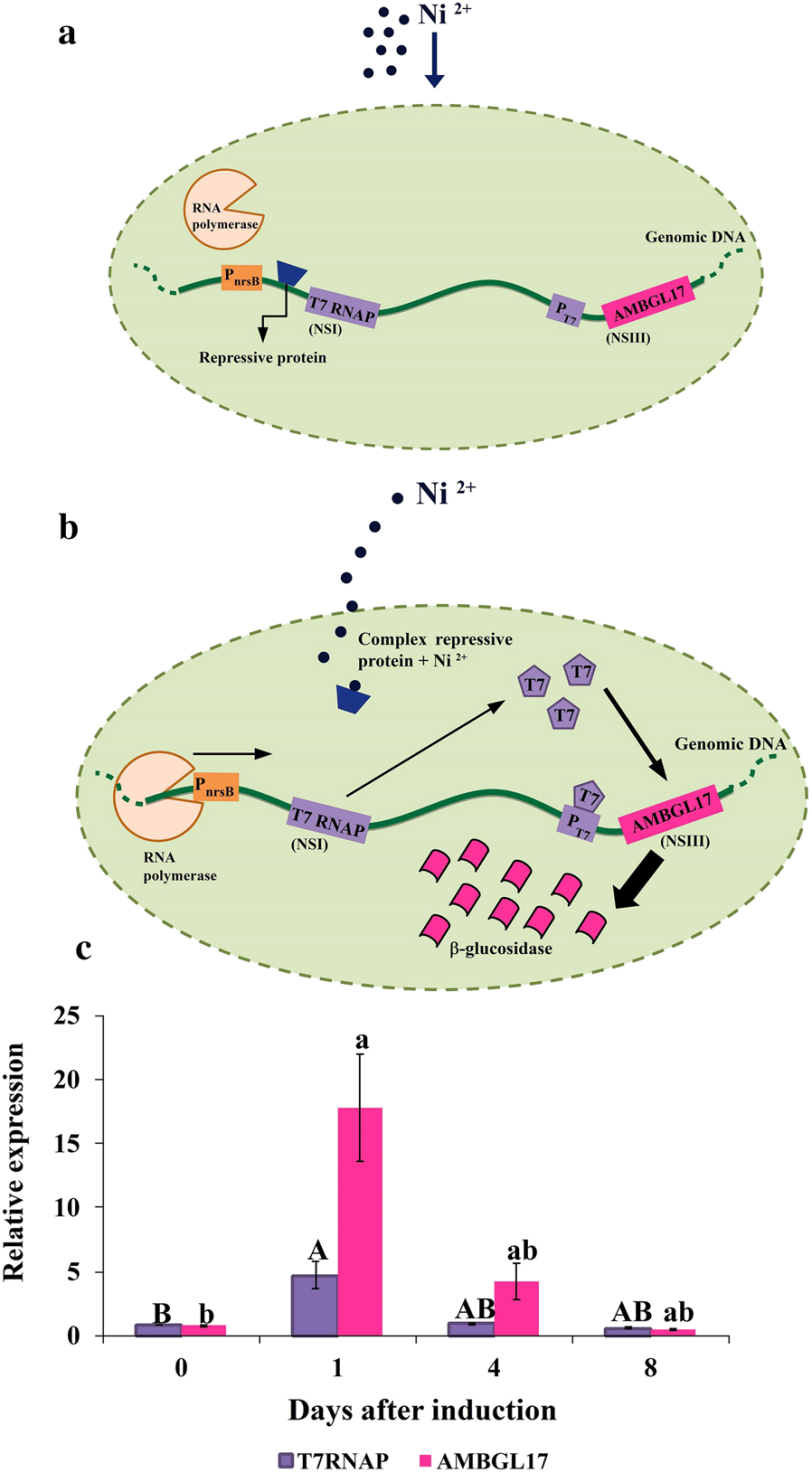
Expression of the T7 system in *Synechococcus elongatus* PCC 7942. **a** Double-transgenic *Synechococcus elongatus* (P_T7_AMBGL17) prior to Ni^2+^ addition. Repressive protein is bound to the DNA and prevents endogenous RNA polymerase interaction with P_nrsB_ that results in T7 RNA polymerase expression. **b** Mechanism of activation of the T7 system in *S. elongatus* P_T7_AMBGL17. After Ni^2+^ addition, this cation binds to the repressive protein, resulting in a change of conformation that blocks its interaction with DNA at the P_nrsB_ region and results in T7RNA polymerase transcription by endogenous RNA polymerase. High levels of expression of T7 RNA polymerase, which action P_T7_ promoter and produces high levels of expression of the AMBGL gene. The thickness of the arrows indicates the intensity of transcription. **c** Relative expression of the T7 RNA polymerase (T7RNAP) and β-glucosidase (AMBGL17) genes in *S. elongatus* P_T7_AMBGL17. The level of gene expression was determined by qPCR, and results were normalized using the constitutive genes rnpB (R-subunit of ribonuclease P) and rpoD (Delta subunit of RNA polymerase). Different letters (uppercase: T7RNAP; lowercase: AMBGL17) show significant statistical differences (*p* < 0.05)

Induction of expression of the target gene in the pET system is based on the use of the P_nrsB_ promoter, which is part of the nrsBACD operon, responsible for cyanobacterium resistance to nickel exposure [26]. When present in the cell, nickel “sequesters” the regulatory protein that blocks interaction between the endogenous RNA polymerase and P_nrsB_ by modifying its conformation, preventing its blocking activity and thus releasing the promoter region, so that transcription of the T7 RNA polymerase gene can start (Fig. 3a, b). The synthesized T7 RNA polymerase binds to its specific promoter (P_T7_) initiating the exclusive transcription of recombinant β-glucosidase (AMBGL17) (Fig. 3b). Although P_nrsB_ is considered a promoter with low non-specific activity, our gene expression data show that in *S. elongatus* the pET system was activated at basal, even without inducer addition to the culture (0 h) (Fig. 3c). This was probably due to the presence of divalent metal ions such as Co^2+^, Zn^2+^ and Cu^2+^ in the BG-11 medium, which are capable of inducing the expression of some promoters, as previously reported to *Synechocystis* sp. PCC 6803, another cyanobacterium species [27]. After 24 h of induction, T7RNAP expression increased approximately 5.5-fold over the uninduced (0 h) timepoint, which was sufficient to raise AMBGL17 gene expression by 22-fold (Fig. 3c).

T7 RNA polymerase is known for its high level of transcription and a low error rate, and thus, relatively small amounts of this enzyme activate the system [19]. The use of the pET system allowed strong gene expression of AMBGL17 in *S. elongatus* PCC 7942, demonstrating that the system is active and efficient. Therefore, in our work, high levels of T7RNAP expression are not required for high expression of the gene of interest, as previously observed [18].

By analyzing the data over the course of days, we observed a decrease in T7RNAP gene expression and, as a consequence, AMBGL17 reaching approximately zero on the eighth day of induction (Fig. 3c). Probably, this occurred because when nickel was added to activate the pET system, the nrsBACD operon was also activated. This operon contains genes encoding proteins that form complex whose function is to pump nickel out of the cell. Therefore, over time, the system was inactivated and AMBGL17 induction stopped.

Englund and Lindberg suggested that one way to prevent the pumping of nickel out of the cell would be to utilize the genes constituting the nrsBACD operon as sites for homologous recombination of the transgene [27]. In this case, the knockout of the nickel efflux system could prolong the expression of the system by potentiating protein production using a lower concentration of the inducer. However, the functionality of the nrsBACD operon remains to be evaluated.

### β-Glucosidase production

Although most enzyme assays involve cell lysis, tests previously performed in our laboratory (data not shown) have shown that the presence of large amounts of phycocyanin pigment present in cyanobacteria lysate interferes with the reading of the colorimetric compound (*para*-nitrophenol—pNP). As the substrate (pNPβG) and enzyme product (pNP) are permeable, being able to cross the cell membrane, it was chosen to carry out the enzymatic activity in the culture medium, without lysing the cells.

**Fig. 4:**
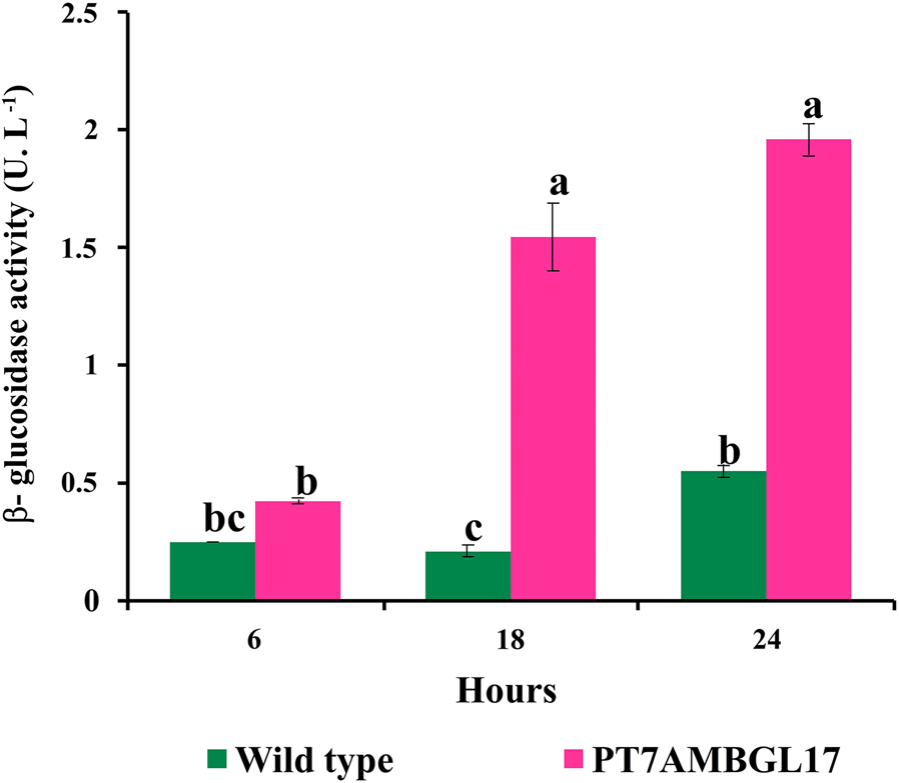
β-glucosidase activity in wild-type and double transgenic lines (P_T7_AMBGL17) at 6, 8 and 24 h using 4-nitrophenyl-β-d-glucopyranoside (pNPβG) as substrate. Different letters show significant statistical differences (*p* < 0.05)

By measuring β-glucosidase activity over 24 h, we observed a significant difference between the *S. elongatus* P_T7_AMBGL17 and wild-type strains (Fig. 4). Three genes coding for potential endogenous β-glucosidases were identified in the genome of *S. elongatus* PCC 7942; these genes are located at loci Synpcc7942_0354, Synpcc7942_0854 and Synpcc7942_1574. It is likely that at least one of these potential β-glucosidases is functional, which would explain the activity observed in the wild-type strain.

In the first 6 h of incubation, β-glucosidase activity was 1.70-fold higher in the transgenic strain compared to wild type, with this difference increasing to 7.4 times after 18 h of incubation, indicating that β-glucosidase activity in transgenic strain would mostly be performed by the heterologous AMBGL17. However, within 24 h this difference decreased to 3.5-fold, probably because most of the substrates (pNPβG) added to the culture were rapidly consumed by the greater amount of β-glucosidases produced by the transgenic strain. This did not occur with the wild strain, which has a lower amount of enzymes, thus taking more time to consume the substrate. If more pNPβG were added to the culture medium, an increase in activity would probably be observed. Several recombinant β-glucosidases have been expressed in bacteria and yeasts [28], but there are still no reports of the use of cyanobacteria as a production platform for this enzyme. The present work demonstrated, for the first time, that it is possible to use cyanobacteria as biofactories to produce recombinant β-glucosidases.

## Conclusions

This is the first work to report an engineered strain of *Synechococcus elongatus* PCC 7942 able to produce β-glucosidase from the pET expression system. Our data show that the pET system is active in the cyanobacterium and promotes high expression of the AMBGL17 gene. It has been shown that the use of an exclusive RNA polymerase for the transcription of heterologous genes can contribute to high levels of transgene expression in cyanobacteria. This strategy can be further applied to the production of other proteins of commercial value. The use of cyanobacteria as biofactories for the production of β-glucosidases could enable the use of CO_2_ and vinasse, effluents of the 1G ethanol plant, as a source of carbon and nitrogen. This could contribute not only to lower production costs, making ethanol 2G a competitive product in the market, but also to integrate 2G production with 1G already existing biorefineries, mitigating the environmental impacts of bioethanol production from sugarcane.

## Materials and methods

### Strains and culture conditions

*Escherichia coli* One Shot TOP10 Electrocomp (Invitrogen) was used for cloning and replication of expression vectors. Bacteria were cultured in Luria–Bertani medium [29] supplemented with 100 μg mL^−1^ spectinomycin, at 37 °C, with constant shaking at 250 rpm*. Synechococcus elongatus* PCC 7942 (Thermo Fisher Scientific) was cultured in BG-11 medium [30], at 34 °C, with constant illumination of 8.000 lx. Transgenic cyanobacterial cultures were supplemented with spectinomycin (10 μg mL^−1^), and expression of the gene of interest was induced with 5 μM NiSO_4_ for 24 h when the absorbance (750 nm) of the cultures reached the value of 1 [31]. Optical density (OD) was monitored in a spectrophotometer (BioMate 3, Thermo Scientific).

### DNA cloning and vector construction

Most important elements of all of the expression vectors used in this work are presented in Table 1. For the production of pSyn1_T7RNAP, the T7 RNA polymerase (T7RNAP) gene was isolated from the *E. coli* BL21 (DE3) genome with the primers T7RNAP-FOR/REV and cloned into pSyn_1 plasmid. The pHN1_T7AMBGL17 vector was constructed by insertion of the β-glucosidase gene (AMBGL17; GenBank: KF433952.1) into the pHN1_lacUV5 vector, amplified by PCR using primers AMBGL17-FOR/REV and PNH1-FOR/REV, respectively. PCR products and plasmids were digested with specific restriction enzymes to enable cloning (pSyn_1 and T7RNAP: *BamH*I and *Kpn*I; pHN1_lacUV5 and AMBGL17: *EcoR*I and *Mlu*I). All enzymes were obtained from New England Biolabs. All DNA fragments were purified (Illustra GFX PCR DNA and gel band purification kit, GE HealthCare) and quantified (Qubit Fluorimeter, Life Technologies), and vectors/inserts were ligated (T7 DNA ligase, New England Biolabs). All procedures were performed following the manufacturer’s protocol, and all genetic constructs were sequenced for confirmation of correct cloning. Table 2 shows the sequences of primers used, which were designed with Primer Express 3.0 software (Applied Biosystems).

**Table 1:** Expression vectors used in this work

Plasmids	Relevant characteristics	Reference
pSyn_1	pUC, Spc^r^, NSI target sites, P_nrsB_, MCS, rrnB	Invitrogen
pHNI_lacUV5	pUC57, Cm^r^, NSIII target sites, MCS, rrnB	[32]
pSyn1_T7RNAP	pUC, Spc^r^, NSI target sites, P_nrsB_, T7 RNAP gene, rrnB	This Study
pHN1_T7AMBGL17	pUC57, Cm^r^, NSIII target sites, P_T7_, AMBGL17 gene, rrnB	This Study

**Table 2:** Sequence of primers used in this work

PRIMERS	Forward primer (5′–3′)	Reverse primer (5′–3′)	Amplicon (bp)
PCR			
T7RNAP	GGATCCGGAAGAGGCACTAAATGAACACG	CCAGGTACCGGAGTCGTATTGATTTGGCG	2697
AMBGL17	CACGAATTCTCGACGCTCTCCCTTATGCGACT	CTGACGCGTTACCGGAAGCAGTGTGACCGTGT	2686
pHN1	CATACGCGTAGGCGGTGAAGGGCAATCAGC	GTAGGAATTCAAGGGCACCAATAACT	3850
Integration			
NSI	TGCTGCGTAACATCGTTGCTGCT	ATGTGATCGGAACCCTGAGCCGT	1400
NSIII	TGGCAGAAGATCGTAGCGGCTCA	CGGATGAGCATTCATCAGGCGGG	1850
qPCR			
RNPB	GTGAGGAGAGTGCCACAGAA	TAAGCCGGGTTCTGTTCTCT	272
RPOD	GAGCAAGCAAGTCAGCGATTTG	TGAGCCCGCAACCACGATC	224
T7RNAP	TTGTCCGGTCGAGGACATCCCTG	CTGATACGGCGAGACTTGCGAGC	153
AMBGL17	GAAAGGCGGTGGCTCTGTGGATG	GCGGCAGGATGTCACCTTCGTTT	104

### Transformation of *S. elongatus*

Transformation of *S. elongatus* PCC 7942 followed the protocol described in the Gene Art *Synechococcus* Engineering kit (Invitrogen). Cyanobacterial strain was first transformed with the pSyn_1T7RNAP vector. After the transgenic confirmation, the strain was again transformed with the second plasmid (pHN1_T7AMBGL17). As a control, *S. elongatus* was also incubated without the expressions vectors. After the transformation process, samples were plated on 1.5% BG-11 agar supplemented with 10 μg mL^−1^ of spectinomycin or 12.5 μg mL^−1^ chloramphenicol.

### Confirmation of genomic integration

PCR was used to confirm the integration of the pSyn1_T7RNAP and pHN1_T7AMBGL17 vectors into the NSI and NSIII sites of the *S. elongatus* genome, respectively. Samples of *S. elongatus* were heated to 95 °C for 5 min, centrifuged briefly and the supernatant used as template for the PCRs. PCRs were performed under the following conditions: 94 °C for 120 s, followed by 35 cycles of 94 °C for 30 s, 58 °C for 30 s, 72 °C for 90 s, and a final extension of 72 °C for 5 min. The primers used are described in Table 2.

### Growth measurement

Inoculum (1.6%) of wild-type and double transgenic (P_T7_AMBGL17) *S. elongatus* cultures were added to erlenmeyers (125 mL) containing 50 mL of BG-11 medium. All cultures were carried out following the conditions described in item 4.1. The cultures used to evaluate the effect of the transgene on growth and addition of nickel to Lag phase cultures started from the same optical density (OD_750_: 0.156 ± 0.003). To evaluate the effect of nickel (Ni^2+^) at the start of the stationary phase, the cultures started from an OD_750_ of 1.047. Growth was monitored in the spectrophotometer (BioMate 3, Thermo Scientific) for 10 days, and 5 μM NiSO_4_ was added at different stages of cultures (OD_750_: 0.156 ± 0.003 and OD_750_: 1.047 ± 0.019) to induce the pET system and evaluate its effect on growth.

### Gene expression analysis

To evaluate expression of the heterologous genes, six samples were collected on days 0 (non-induced), 1, 4 and 8 after induction with 5 μM NiSO_4_. Total RNA was extracted with Trizol (Invitrogen) and treated with DNAse I (Invitrogen). The cDNA synthesis was performed with the High Capacity cDNA Reverse Transcription kit (Applied Biosystems), and cDNA was quantified using the Qubit fluorimeter (Invitrogen). Gene expression was analyzed by quantitative real-time PCR (qPCR) using Platinum SYBR Green qPCR SuperMix-UDG (Invitrogen). Serial dilutions of cDNA were performed to test the efficiency of all primers. The conditions of qPCRs were 50 °C for 120 s, 95 °C for 120 s, followed by 40 cycles of 95 °C for 15 s and 60 °C for 30 s. The expression of the target genes was normalized by the constitutive genes *rnpB* and *rpoD* [33]. All procedures were performed following the manufacturer’s protocol, and the primers used are described on Table 2. Data from qPCR were analyzed by the delta C_T_ method [34].

### β-Glucosidase activity

β-Glucosidase activity was determined by using p-nitrophenyl-β-d-glucopyranoside substrate (pNPβG) as described by Strahsburger et al. [35], who performed in vivo assays of β-glucosidase activity on strains of *Bifidobacterium* and *Lactobacillus* by incubating microorganisms with pNPβG. After 24 h of induction with 5 μM NiSO_4_, 1.600 μg mL^−1^ of pNPβG was added to wild-type and transgenic *S. elongatus* cultures. Culture samples were collected for the determination of total protein concentration (Qubit Fluorimeter, Life Technologies) and β-glucosidase activity after 6, 18 and 24 h of the addition of pNPβG. Cells were precipitated by centrifugation at 10,000*g* for 5 min. The supernatant (150 μL) was transferred to a clear 96-well plate containing 50 μL of 0.1 M Na_2_CO_3_. Absorbance was measured at the wavelength of 405 nm (BioTek ELx800 Microplate Reader). A unit (U) of enzymatic activity is defined as the amount of β-glucosidase required to release 1 μmol of pNPβG per minute per gram of protein under the assay conditions.

### Statistical analysis

For gene expression analysis, it was used six samples for each treatment. Growth measurement and β-glucosidase activity were performed in triplicate. One-way ANOVA was used to evaluate expression of T7RNAP and AMBGL17 genes over time. The effect of transgenesis and nickel addition on the growth of *S. elongatus* PCC 7942 was evaluated by two-way ANOVA with repeated measures (factors: transgenesis and time/nickel and time). The effect of transgenesis on enzymatic activity was analyzed by two-way ANOVA with repeated measures (factors: transgenesis and time). All data were tested for their normality and homoscedasticity using the Shapiro–Wilk and Bartlett tests, respectively. A *p* < 0.05 was considered, and all the statistical analyses were performed with R Software [36].

## Data Availability

The datasets used and/or analyzed during the current study are available from the corresponding author on reasonable request.

## Abbreviations

1G: first generation
2G: second generation
CO_2_: carbon dioxide
Mt: millions of tons
NSI: neutral site 1
NSIII: neutral site 3
PCR: polymerase chain reaction
Ni ^2+^: nickel
T7RNAP: T7 RNA polymerase
AMBGL17: β-glucosidase AMBGL17
Zn^2+^: zinc
Cu^2+^: copper
Co^2+^: cobalt
pNPβG: *p*-nitrophenyl-β-d-glucopyranoside
P_T7_: T7 promoter
P_nrsB_: nickel-inducible promoter
NiSO_4_: nickel sulfate
Na_2_CO_3_: sodium carbonate
OD: optical density
qPCR: quantitative real-time PCR
